# Spatiotemporal Accumulation of Four Major Bioactive Compounds in *Zanthoxylum nitidum* Roots: Implications for Tissue-Specific Processing and Harvesting Strategies

**DOI:** 10.3390/plants15203101

**Published:** 2026-10-10

**Authors:** Tao Tian, Sha Gao, Yi Wu, Siyan Tan, Sisheng Zhang, Hong Wu, Yang Yang

**Affiliations:** 1 School of Pharmaceutical Sciences, Hunan Medical College, Huaihua 418000, China; 2 Hunan Provincial Key Laboratory for Synthetic Biology of Traditional Chinese Medicine, Hunan Medical College, Huaihua 418000, China; 3 State Key Laboratory for Conservation and Utilization of Subtropical Agro-Bioresources, South China Agricultural University, Guangzhou 510642, China

**Keywords:** *Zanthoxylum nitidum*, tissue specificity, age-dependent accumulation, harvesting age, chemometric analysis

## Abstract

This study investigated the spatial distribution and age-dependent accumulation of major bioactive compounds in cultivated *Zanthoxylum nitidum* roots to optimize harvesting strategies and quality evaluation. The root bark (RB) radial proportion increased during early growth stages, peaking at 16.67% in five-year-old plants. All four compounds exhibited pronounced tissue specificity, preferentially accumulating in RB, whereas root wood (RW), accounting for 83.33–93.67% of total root radial proportion, maintained low concentrations. The accumulation patterns of individual compounds varied markedly with growth age: nitidine chloride (NC) and chelerythrine (CHE) reached their highest concentrations in three-year-old RB (0.89% and 2.22%), magnoflorine (MAG) showed a bimodal pattern with peaks in the first and fifth years, and hesperidin (HES) displayed a distinct V-shaped trend. Hierarchical cluster analysis (HCA) and principal component analysis (PCA) consistently demonstrated that tissue type was the primary source of variation in chemical composition, whereas age-related differences within RB were mainly associated with HES, NC, and CHE. These findings provide valuable preliminary guidance on tissue-specific accumulation and structural development, offering a framework for refining harvesting strategies and quality evaluation.

## 1. Introduction

*Zanthoxylum nitidum* (Roxb.) DC., belonging to the family Rutaceae, is a perennial woody climbing vine. It is mainly distributed in southern China, Vietnam, Laos, India, Indonesia, New Guinea, and Australia [1,2]. In China, the dried roots of *Z. nitidum* are widely used as medicinal materials and are traditionally applied for promoting blood circulation, relieving pain, dispelling wind-dampness, dredging meridians, and reducing swelling and toxicity [3]. Currently, *Z. nitidum* serves as an important raw material for more than 60 traditional Chinese patent medicines and has shown considerable application prospects in pharmaceutical and personal care industries [3,4]. However, continuous overharvesting of wild populations has resulted in severe depletion of natural resources, making artificial cultivation an essential strategy for sustainable utilization of this medicinal plant [4,5,6].

The medicinal properties of *Z. nitidum* roots are mainly associated with a diverse array of bioactive compounds, particularly alkaloids, and flavonoids [7,8]. Among them, nitidine chloride (NC), a benzophenanthridine alkaloid, is the only quality marker officially specified by the *Pharmacopoeia of the People’s Republic of China* (PPRC), with a minimum required content of 0.13% [3]. In addition, there exist several other pharmacologically active constituents, including chelerythrine (CHE), magnoflorine (MAG), and hesperidin (HES). CHE and MAG serve as key active alkaloids with proven synergistic anti-inflammatory and analgesic efficacy, whereas HES represents the dominant flavonoid responsible for antioxidant capabilities in Rutaceae species [2,7,9,10]. Therefore, comprehensive quality evaluation of *Z. nitidum* usually requires the simultaneous assessment of multiple bioactive compounds to better characterize the overall medicinal quality of cultivated materials.

The biosynthesis and accumulation of secondary metabolites in medicinal plants represent dynamic processes regulated by developmental stage and tissue specificity [11,12]. In the roots of perennial medicinal plants, bioactive compounds often exhibit distinct spatial distribution patterns between root bark (RB) and root wood (RW) [13,14,15]. As plants age, the proportion of RW generally increases substantially, which may alter the chemical profile of the entire medicinal material and dilute the concentration of target compounds [16,17]. Although previous studies have investigated geographical variation and essential oil composition of *Z. nitidum* [18,19], the spatial distribution and dynamic accumulation patterns of these four key bioactive compounds in RB and RW across different growth ages (1–6 years) remain poorly understood. This knowledge gap has hindered the precise utilization of specific medicinal tissues, optimization of harvesting strategies, and breeding of high-quality cultivars of *Z. nitidum*.

To address these issues, roots of cultivated *Z. nitidum* plants aged 1–6 years were collected from Yunfu City, Guangdong Province, China. High-performance liquid chromatography (HPLC), combined with hierarchical cluster analysis (HCA) and principal component analysis (PCA), was employed to systematically evaluate tissue-partitioned (RB vs. RW) dynamic accumulation across a complete 1–6 year developmental continuum. This study aimed to (1) elucidate the dynamic accumulation patterns of these bioactive compounds during different growth stages; (2) reveal the differential distribution of chemical constituents between RB and RW; and (3) determine the optimal harvesting age based on integrated chemical profiling and chemometric evaluation. The findings provide a scientific basis for quality standardization, rational harvesting, and efficient utilization of *Z. nitidum* resources.

## 2. Materials and Methods

### 2.1. Experimental Materials

**Figure 1:**
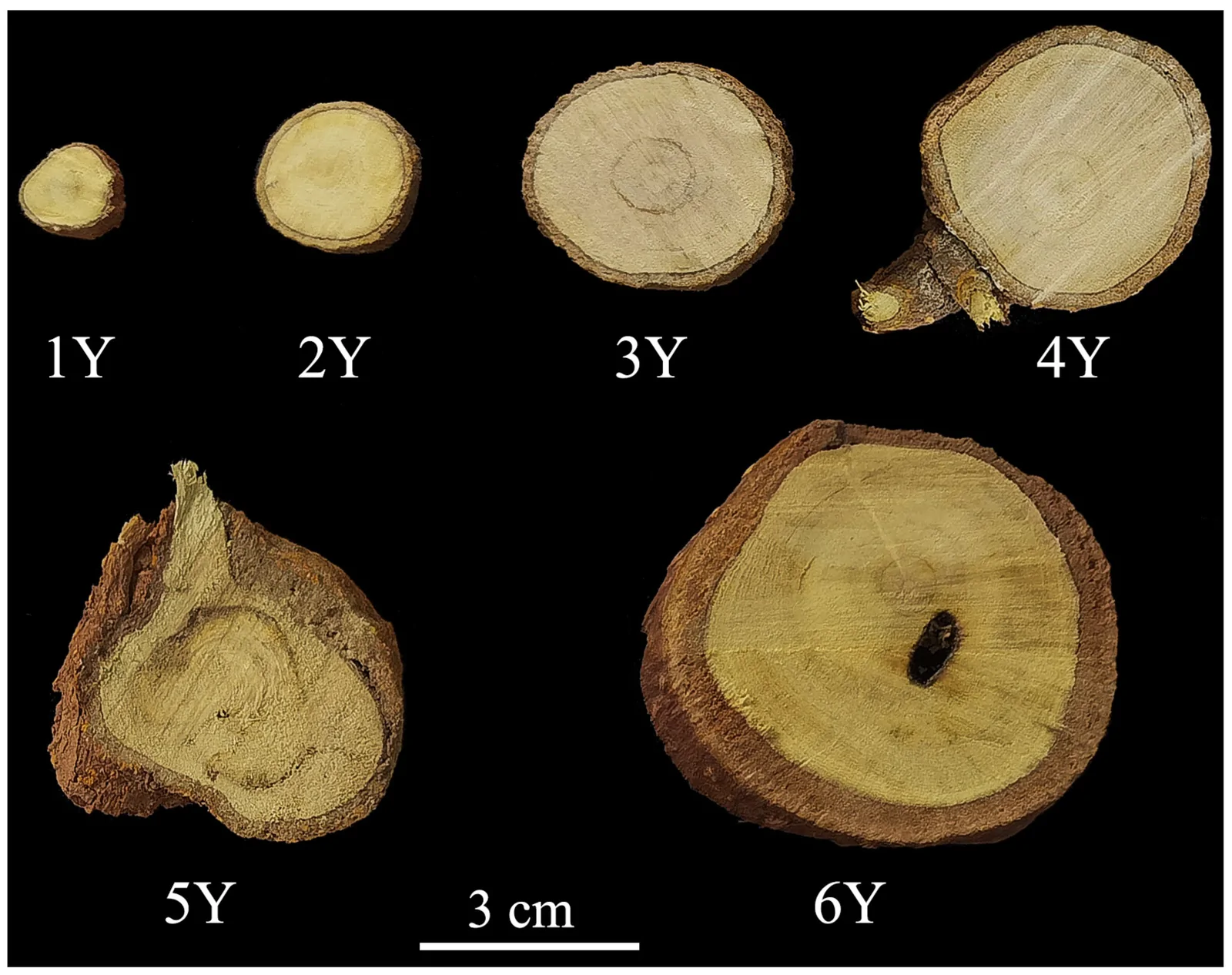
Cross-sections of *Zanthoxylum nitidum* roots at different growth years, showing the morphological differences in root diameter and bark thickness. 1Y–6Y denote the roots of *Z. nitidum* at 1–6 years of growth, respectively. Bar = 3 cm.

Plant materials were collected in November 2022 from a standardized cultivation base of *Z. nitidum* located in Yunfu City, Guangdong Province, China (111°03′ E, 22°22′ N). Cultivated plants aged 1–6 years were identified as *Z. nitidum* (Rutaceae) by Professor Hong Wu of South China Agricultural University and Associate Professor Yang Yang of Hunan Medicine College (Figure 1). For each growth age, nine healthy plants with uniform growth and free of visible diseases or insect damage were randomly selected, and the primary root of each individual was harvested. For each age group, nine plants were randomly assigned to three groups (3 plants per group) and pooled to create three independent biological replicates (n = 3) for tissue preparation and chemical analysis. Fresh roots were thoroughly washed with tap water and separated along the vascular cambium into RB (including the periderm and phloem; RB1Y–RB6Y) and RW (xylem; RW1Y–RW6Y). The separated tissues were air-dried, ground into powder, passed through a 60-mesh sieve, and stored in sealed containers in a cool, dry place until chemical analysis. Voucher specimens (Nos. ZN-20221106-01 to ZN-20221106-06) were deposited in the medicinal plant research center of South China Agricultural University.

### 2.2. Determination of the Radial Proportions of RB and RW

Root samples from different growth ages were used to determine root diameter (mm) and RB thickness (mm) with a digital vernier caliper (Deli Group Co., Ltd., Ningbo, China). Cross-sections were taken from the primary root at 3 cm above the ground surface. The thickness of RB and the radius of RW were measured along two mutually perpendicular directions on each cross-section. The mean of nine measurements was used to represent the radial proportion of each tissue for each growth age. The radial proportions of RB and RW were calculated as follows:

RB radial proportion (%) = RB thickness/Root radius × 100

RW radial proportion (%) = RW radius/Root radius × 100

### 2.3. Extraction of Bioactive Compounds

Approximately 0.50 g of powdered sample was accurately weighed into a stoppered conical flask and extracted with 25 mL of 70% methanol by ultrasonic-assisted extraction (300 W, 40 kHz) for 60 min. After cooling to room temperature, the extract was filtered, and the filtrate was collected in a 50 mL volumetric flask. The residue, together with the filter paper, was returned to the conical flask and extracted again with an additional 25 mL of 70% methanol under the same ultrasonic conditions for another 60 min. After filtration, the two filtrates were combined. The flask and residue were rinsed twice with a small volume of 70% methanol, and the rinsing solutions were added to the combined extract. The final volume was adjusted to 50 mL with 70% methanol and mixed thoroughly. The solution was filtered through a 0.22 μm membrane filter, and the filtrate was stored at 4 °C in the dark until HPLC analysis. All constituent contents were calculated based on the initial dry tissue weight (% *w*/*w*). Sample extractions were conducted in triplicate using the three independent biological replicates.

### 2.4. HPLC Analysis

The HPLC method was adopted from the PPRC [3]. Chromatographic analysis was performed using an Agilent 1260 HPLC system equipped with a diode-array detector (DAD) (Agilent Technologies, Santa Clara, CA, USA). Separation was achieved on an Agilent ZORBAX SB-C18 column (4.6 × 250 mm, 5 μm) (Agilent Technologies, Santa Clara, CA, USA). The column temperature was maintained at 30 °C, the flow rate was set at 1.00 mL·min^−1^, the detection wavelength was 273 nm, and the injection volume was 10 μL. The mobile phase consisted of solvent A (acetonitrile) and solvent B (0.1% formic acid solution adjusted to pH 4.5 with triethylamine). The gradient elution program was as follows: 0–3 min, 20% A; 3–33 min, 20–50% A; 33–40 min, 50–95% A; and 40–45 min, 95–20% A.

Reference standards of nitidine chloride (CAS No. 13063-04-2), chelerythrine (CAS No. 34316-15-9), magnoflorine (CAS No. 2141-09-5), and hesperidin (CAS No. 520-26-3) were purchased from Chengdu Must Bio-Technology Co., Ltd. (Chengdu, China). Calibration curves for each compound were established using five standard solutions of different concentrations, with peak area (y) plotted against analyte concentration (x). Linear regression analysis generated the calibration equations, and all compounds exhibited excellent linearity over the tested concentration ranges (R^2^ > 0.999) (Figure S1).

### 2.5. Hierarchical Cluster Analysis (HCA)

The contents of the major bioactive compounds in RB and RW of *Z. nitidum* at different growth ages were standardized using Z-score normalization before analysis. The standardized dataset was imported into SPSS 20.0 (IBM Corp., Armonk, NY, USA) for HCA. Clustering was performed using the between-groups linkage method with squared Euclidean distance as the similarity measure. To simultaneously evaluate tissue specificity and growth age effects, two HCA models were constructed: an overall HCA including all 12 samples (RB and RW from six growth ages) and an independent HCA including only the six RB samples from different growth ages. Distance matrices were generated, and the clustering results were visualized as dendrograms.

### 2.6. Principal Component Analysis (PCA)

The contents of the major bioactive compounds in RB and RW of *Z. nitidum* at different growth ages were standardized using Z-score normalization prior to PCA. The standardized data were analyzed using OriginPro 2024 (OriginLab Corporation, Northampton, MA, USA). Principal components with eigenvalues greater than 1 were retained, and score plots based on the first two principal components (PC1 and PC2) were generated. To simultaneously evaluate tissue specificity and growth age effects, PCA was performed for both the complete dataset comprising all 12 samples and the subset consisting of the six RB samples. Loading plots were subsequently generated to determine the contribution of each bioactive compound to the principal components.

### 2.7. Data Analysis

Data are presented as Mean ± SD (n = 3 for chemical assays; n = 9 for radial measurements). Two-way ANOVA was performed to examine main effects of tissue type, plant age, and their interaction. One-way ANOVA followed by Duncan’s test was used for comparisons among ages within each tissue type. *p* < 0.05 was considered statistically significant.

## 3. Results

### 3.1. Changes in the Radial Proportions of RB and RW at Different Growth Ages

**Figure 2:**
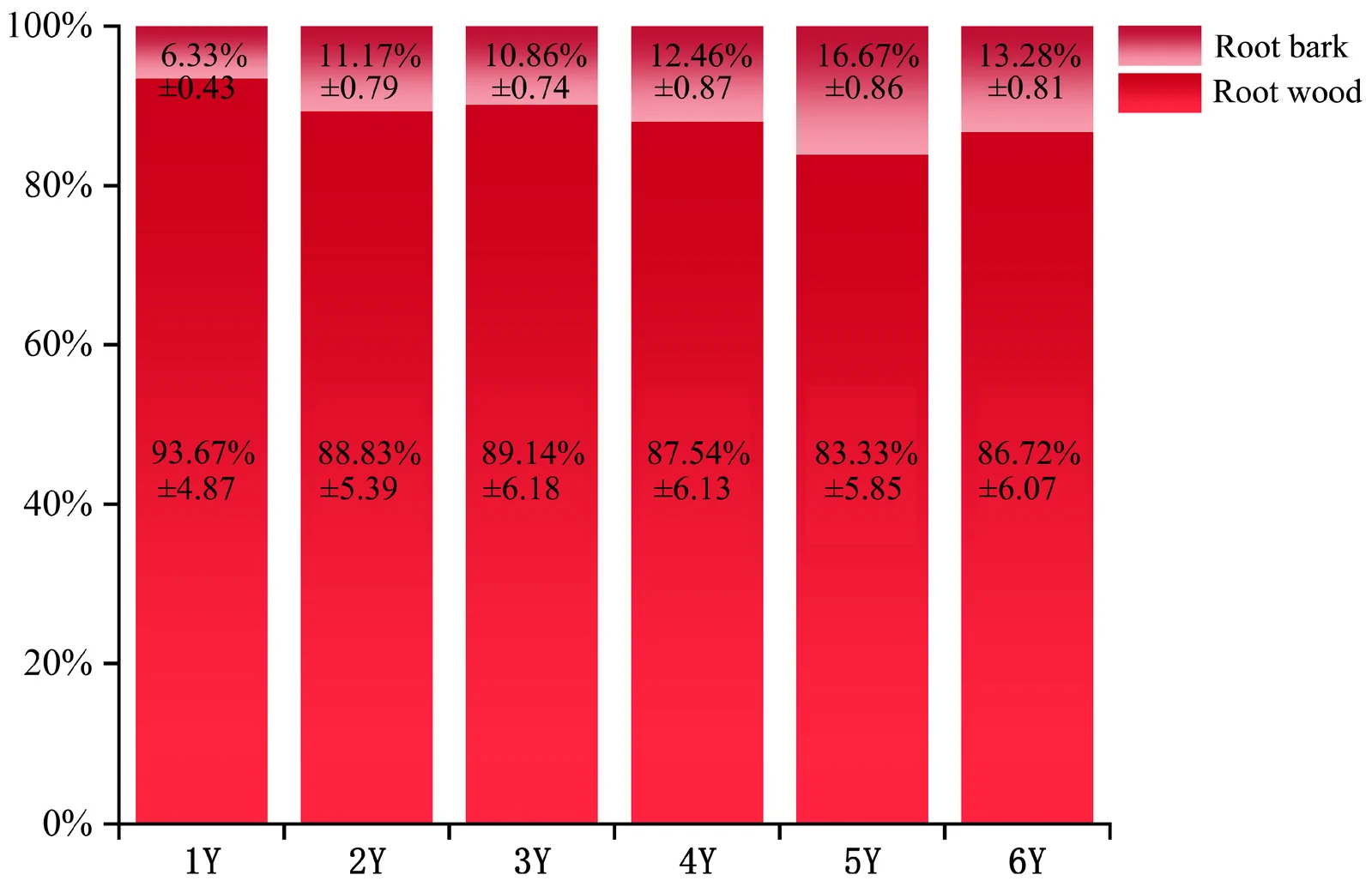
Radial proportions of root bark (RB) and root wood (RW) in *Zanthoxylum nitidum* roots at different growth years. 1Y–6Y denote the roots of *Z. nitidum* at 1–6 years of growth, respectively. Mean ± SD (n = 9).

The radial proportion of RW in *Z. nitidum* exhibited an overall pattern of an initial decrease followed by a slight increase with increasing growth age (Figure 2). The highest radial proportion of RW was observed in one-year-old plants (93.67%). Thereafter, the radial proportions in two- to six-year-old plants were 88.83%, 89.14%, 87.54%, 83.33%, and 86.72%, respectively, with the lowest value recorded in five-year-old plants (83.33%). Conversely, the radial proportion of RB showed an overall trend of increasing initially and then decreasing with plant age (Figure 2). The radial proportions of RB in one- to five-year-old plants were 6.33%, 11.17%, 10.86%, 12.46%, and 16.67%, respectively, reaching the maximum value in the fifth year before declining slightly to 13.28% in six-year-old plants.

### 3.2. Contents of Major Bioactive Compounds in the RB of Z. nitidum at Different Growth Ages

**Figure 3:**
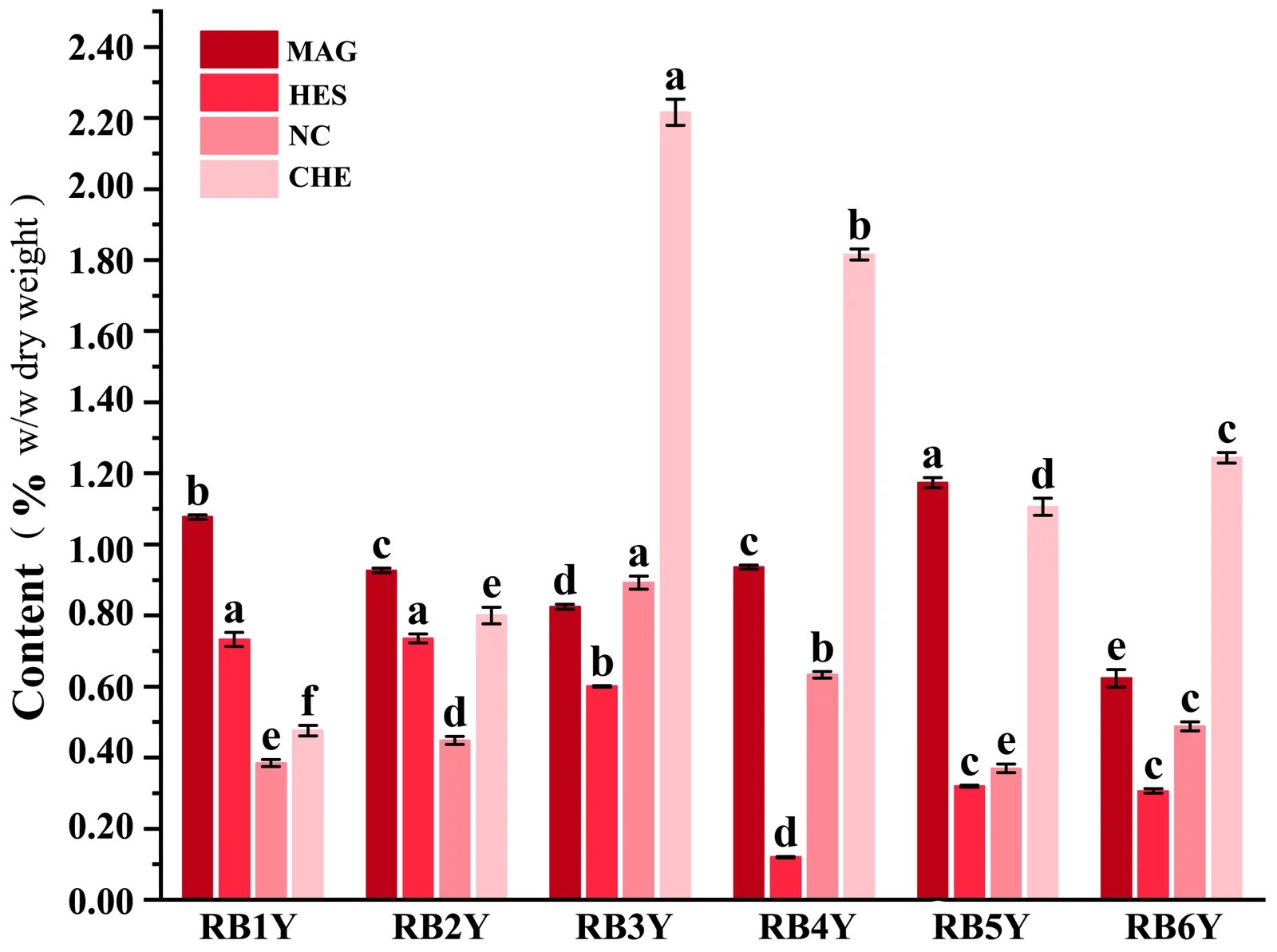
Content of four active compounds in the root bark (RB) of *Zanthoxylum nitidum* at different growth years. RB1Y–RB6Y denote the RB samples collected from 1–6-year-old *Z. nitidum* plants, respectively. MAG: magnoflorine, HES: hesperidin, NC: nitidine chloride, CHE: chelerythrine. Different lowercase letters “a, b, c, d, e, f” above the bars indicate statistically significant differences among years for the same compound (*p* < 0.05). Data are expressed as Mean ± SD (n = 3 independent biological replicates).

As shown in Figure 3, the NC content in the RB of *Z. nitidum* ranged from 0.37% to 0.89% across different growth ages, and all samples exceeded the minimum requirement of the PPRC (≥0.13%) [3]. The NC content generally increased during the early growth stages and declined thereafter, reaching the highest level in RB3Y (0.89%), which was significantly higher than those of the other growth ages (*p* < 0.05). A similar accumulation pattern was observed for CHE, with its content increasing initially and then decreasing. The highest CHE content was detected in RB3Y (2.22%), which was significantly higher than those of the other samples (*p* < 0.05). In contrast, MAG exhibited a bimodal accumulation pattern, with two local maxima occurring in RB1Y (1.08%) and RB5Y (1.17%). Among them, the MAG content in RB5Y was significantly higher than those of all other growth ages (*p* < 0.05). The HES displayed a distinct V-shaped trend with increasing growth age. Its lowest content was recorded in RB4Y (0.12%), whereas significantly higher levels were detected in RB1Y (0.73%) and RB2Y (0.73%) than in the remaining growth ages (*p* < 0.05).

### 3.3. Contents of Major Bioactive Compounds in the RW of Z. nitidum at Different Growth Ages

**Figure 4:**
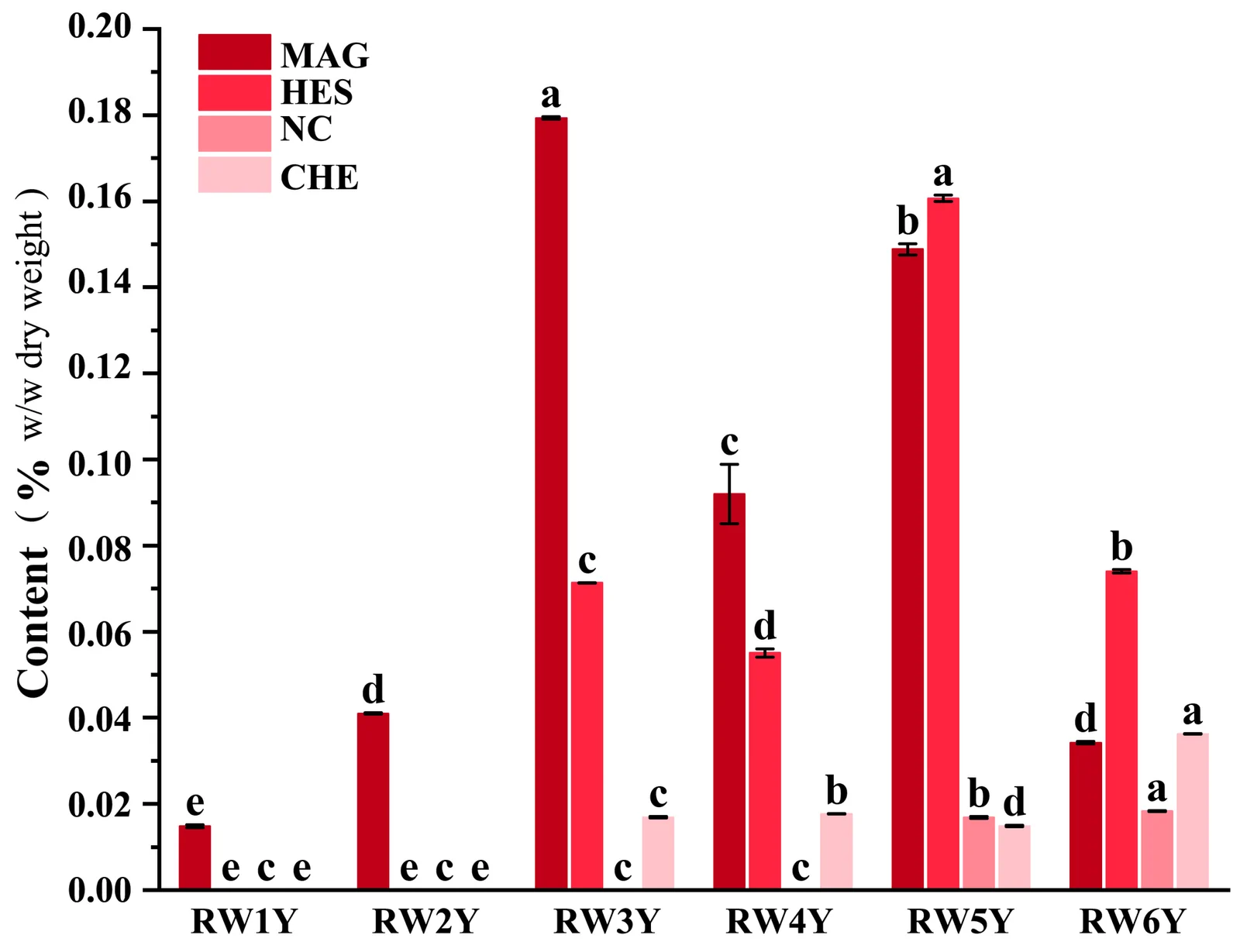
Content of four active compounds in the root wood (RW) of *Zanthoxylum nitidum* at different growth years. RW1Y–RW6Y denote the RW samples collected from 1–6-year-old *Z. nitidum* plants, respectively. MAG: magnoflorine, HES: hesperidin, NC: nitidine chloride, CHE: chelerythrine. Different lowercase letters “a, b, c, d, e” above the bars indicate statistically significant differences among years for the same compound (*p* < 0.05). Data are expressed as Mean ± SD (n = 3 independent biological replicates).

Compared with RB (Figure 3), the contents of all four bioactive compounds in RW were significantly lower (*p* < 0.05) (Figure 4). The NC content was extremely low in RW1Y–RW4Y. In RW5Y and RW6Y, the NC contents were only 0.017% and 0.018%, respectively, both well below the minimum requirement of the PPRC (≥0.13%) [3]. CHE was not detected in RW1Y or RW2Y (LOD = 0.08 μg/mL). The highest CHE content was observed in RW6Y (0.036%), which was significantly higher than in RW3Y–RW5Y (*p* < 0.05). Similarly, HES was not detected in RW1Y or RW2Y (LOD = 0.12 μg/mL). The HES content reached its maximum in RW5Y (0.16%), which was significantly higher than those of the other growth ages (*p* < 0.05). MAG was the predominant bioactive compound in RW, with contents ranging from 0.015% to 0.18%. Its concentration reached a maximum in RW3Y (0.18%) and exhibited a secondary peak in RW5Y (0.15%), indicating a bimodal accumulation pattern. Overall, RW contained substantially lower concentrations of bioactive compounds than RB, suggesting that it contributes relatively little to the medicinal quality of *Z. nitidum* roots when considered independently.

### 3.4. HCA of Major Bioactive Compounds in Z. nitidum Roots at Different Growth Ages

**Figure 5:**
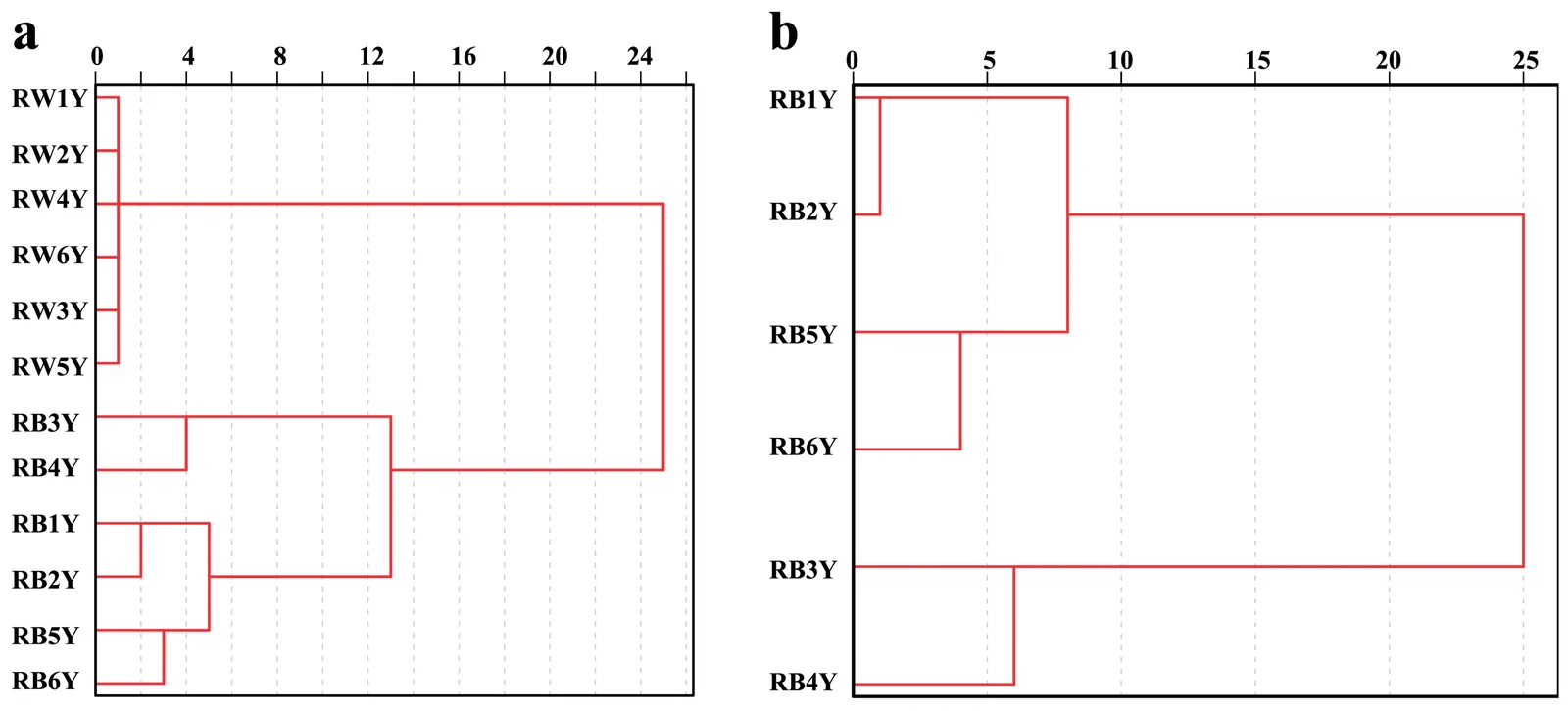
Hierarchical cluster analysis (HCA) of (**a**) root bark (RB) and root wood (RW), (**b**) RB. RB1Y–RB6Y and RW1Y–RW6Y denote the RB and RW samples, respectively, collected from *Zanthoxylum nitidum* at 1–6 years of growth. The horizontal axis represents the Euclidean distance.

The overall HCA results showed that, at a squared Euclidean distance of approximately 13–25, the 12 samples were separated into two major clusters (Figure 5a). All RW samples (RW1Y–RW6Y) were grouped into one cluster, whereas all RB samples (RB1Y–RB6Y) formed the other cluster. This clear separation between RB and RW indicates that tissue type is the primary source of variation in the chemical composition. At a squared Euclidean distance of approximately 5–13, the six RB samples were further divided into two subclusters. One subcluster consisted of RB3Y and RB4Y, whereas the other comprised RB1Y, RB2Y, RB5Y, and RB6Y. In contrast, all RW samples remained clustered together even at a squared Euclidean distance of ≥1, indicating a high degree of similarity in their chemical composition across different growth ages. To eliminate the influence of tissue type, HCA was further performed using only the RB samples (Figure 5b). At a squared Euclidean distance of approximately 6, the six RB samples were classified into three distinct clusters: RB1Y–RB2Y, RB3Y–RB4Y, and RB5Y–RB6Y. This clustering pattern was consistent with that obtained from the overall HCA.

### 3.5. PCA of Major Bioactive Compounds in Z. nitidum Roots at Different Growth Ages

**Figure 6:**
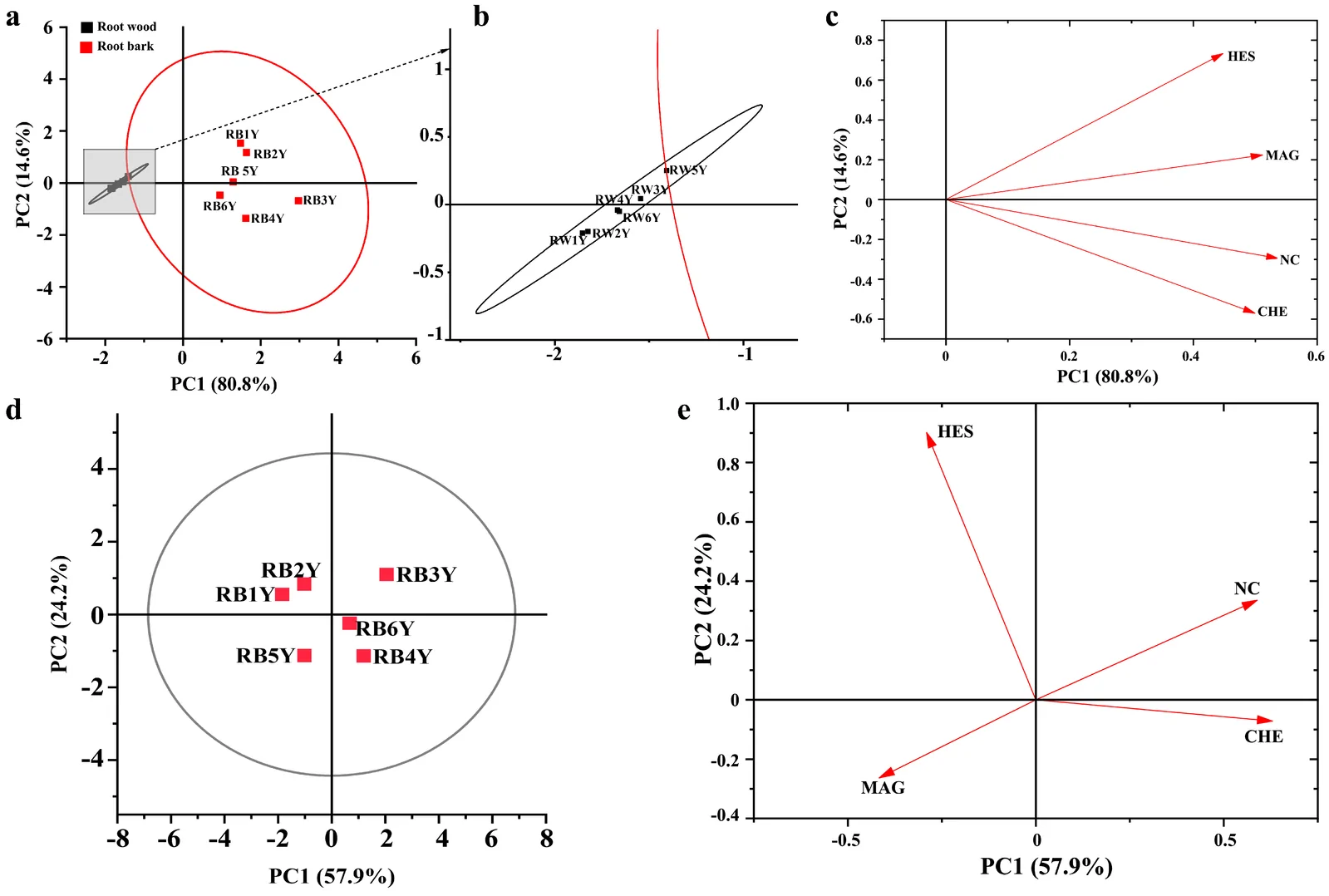
Principal component analysis (PCA) score plots and loading plots of *Zanthoxylum nitidum* samples. (**a**) PCA score plots of root bark (RB) and root wood (RW); (**b**) enlarged PCA scoring chart of RW; (**c**) PCA loading plots of RB and RW; (**d**) PCA score plots of RB; (**e**) PCA loading plots of RB. RB1Y–RB6Y and RW1Y–RW6Y denote the RB and RW samples, respectively, collected from *Zanthoxylum nitidum* at 1–6 years of growth. MAG: magnoflorine, HES: hesperidin, NC: nitidine chloride, CHE: chelerythrine. The ellipses in the figure represent confidence regions reflecting the degree of intra-group clustering and inter-group separation. The arrows indicate the loading vectors, pointing in the direction and magnitude of the contribution of each compound. A longer vector signifies a greater influence, whereas the angle represents the correlation between the component and the samples.

PCA of all 12 samples showed that PC1 explained 80.80% of the total variance, whereas PC2 accounted for 14.60%, resulting in a cumulative contribution of 95.40% (Figure 6a). The six RB samples (red symbols) were distributed along the positive side of PC1 with relatively dispersed clustering, whereas the six RW samples (black symbols) formed a compact cluster on the negative side of PC1, indicating that PC1 primarily reflected tissue-specific differences. The enlarged PCA score plot of the RW samples (Figure 6b) showed a high degree of overlap among different growth ages, indicating that the chemical composition of RW varied only slightly with plant age. This result was consistent with the HCA dendrogram. The loading plot (Figure 6c) showed that all four compounds exhibited relatively high positive loadings on PC1, ranging from 0.45 to 0.54. HES and MAG showed positive loadings on PC2, with HES exhibiting the highest loading (0.73), whereas NC and CHE showed negative loadings of −0.29 and −0.57, respectively. The overall contribution scores of HES, MAG, NC, and CHE were 0.24, 0.22, 0.25, and 0.25, respectively. These results suggest that NC, CHE, and HES are the principal chemical markers distinguishing RB from RW.

PCA of the six RB samples showed that PC1 and PC2 explained 57.90% and 24.20% of the total variance, respectively, with a cumulative contribution of 82.10% (Figure 6d). RB3Y, RB4Y, and RB6Y were distributed on the positive side of PC1, whereas RB1Y, RB2Y, and RB5Y were located on the negative side. Along PC2, RB1Y–RB3Y were distributed on the positive side, whereas RB4Y–RB6Y were positioned on the negative side. Overall, RB1Y and RB2Y formed one subgroup, and RB4Y and RB6Y formed another subgroup, whereas RB3Y and RB5Y each occupied distinct positions. In the loading plot for the RB samples (Figure 6e), NC and CHE exhibited positive loadings on PC1 (0.59 and 0.63, respectively), whereas HES and MAG showed negative loadings (−0.29 and −0.42, respectively). On PC2, HES and NC had positive loadings of 0.90 and 0.34, respectively, whereas MAG and CHE had negative loadings of −0.26 and −0.07. The overall contribution scores of HES, MAG, NC, and CHE were 0.25, 0.12, 0.23, and 0.23, respectively. These results indicate that age-related variation in the chemical composition of RB was primarily associated with HES, NC, and CHE, which is consistent with the quantitative analysis showing that the accumulation of these compounds reached its highest levels during the third and fourth years of growth.

## 4. Discussion

### 4.1. Tissue-Specific Distribution of Bioactive Compounds in Z. nitidum

The present study demonstrated that all four major bioactive compounds were preferentially accumulated in the RB of *Z. nitidum*, whereas the concentration of NC in RW remained far below the minimum requirement specified by the PPRC [3]. Furthermore, both HCA and PCA consistently separated all samples into two distinct groups corresponding to RB and RW, indicating that tissue type is the principal factor contributing to variation in chemical composition.

These findings are consistent with previous pharmacological studies. Han et al. [20] reported that RB exhibited stronger analgesic and gastroprotective activities than RW. Likewise, the antibacterial, anti-inflammatory, and analgesic activities of *Z. nitidum* roots and stems have been shown to be predominantly associated with bark tissues [21,22]. The present study provides quantitative chemical evidence supporting these pharmacological observations.

Tissue-specific accumulation of secondary metabolites is not unique to *Z. nitidum* but has also been widely reported in other medicinal plants. In *Taxus media* Rehder, paclitaxel, 10-deacetylbaccatin III (10-DAB), and 3′-*N*-debenzoyltaxol (DAP) are predominantly accumulated in the stem phloem, whereas their concentrations are lowest in the xylem [23]. In *Salvia miltiorrhiza* Bunge, tanshinones and salvianolic acids are mainly localized in the cork tissues of the root [24]. Similarly, in *Coptis chinensis* Franch, palmatine is mainly distributed in the pith, whereas oxyberberine is concentrated in the cork and xylem rays [25]. Collectively, these studies indicate that secondary metabolites commonly exhibit pronounced tissue specificity, which is closely associated with vascular differentiation, the distribution of storage cells, and the spatial expression of biosynthetic enzymes. Because RW contained only trace amounts of the quantified bioactive compounds while accounting for more than 80% of the root radial proportion, inclusion of the entire root may reduce the overall concentration of these constituents through a dilution effect. Therefore, RB represents the principal tissue contributing to the chemical quality of cultivated *Z. nitidum* medicinal materials.

### 4.2. Growth Age Significantly Influences the Dynamic Accumulation of Major Bioactive Compounds

The biosynthesis and accumulation of plant secondary metabolites are continuously regulated throughout plant development. In the present study, the four major bioactive compounds exhibited distinct accumulation patterns across different growth ages, indicating that different classes of metabolites follow different developmental trajectories. Benzophenanthridine alkaloids (NC and CHE) reached their maximum concentrations in the third year, and MAG displayed a bimodal accumulation pattern, whereas the flavonoid HES exhibited a characteristic V-shaped trend.

These patterns are consistent with the Growth–Differentiation Balance Hypothesis (GDBH) [26,27], which proposes that plants allocate available resources differently among growth, differentiation, and secondary metabolism during development [19,28]. During the early stages of growth, resources are preferentially invested in organ development and primary metabolism, while relatively high levels of flavonoids, such as HES, contribute to stress resistance and defense in young tissues [28,29]. As vegetative growth slows with plant maturation, metabolic flux is redirected toward phenylalanine-derived and isoquinoline alkaloid biosynthetic pathways, promoting the rapid accumulation of NC and CHE in the RB of three-year-old plants [28,30,31]. During later developmental stages, physiological senescence, progressive lignification, and competition among metabolic pathways may contribute to the decline or redistribution of certain secondary metabolites [28,32].

Comparable age-dependent accumulation patterns have been reported in other perennial medicinal plants. In *Scutellaria baicalensis* Georgi, the optimal balance between bioactive compound content and annual biomass production was achieved in two- to three-year-old roots, whereas the increase in biomass became markedly lower after the third year despite relatively high concentrations of some constituents [33]. Likewise, a distinct temporal relationship between biomass accumulation and alkaloid production has been reported in stem cultures of *Catharanthus roseus* (L.) G. Don [26]. These findings suggest that extending the cultivation period does not necessarily maximize the production of target metabolites, emphasizing the importance of determining harvesting time according to the accumulation dynamics of specific bioactive compounds.

### 4.3. Changes in the Radial Proportion of RB May Influence the Overall Chemical Quality of Medicinal Roots

The chemical quality of perennial woody medicinal plants is determined not only by the concentration of bioactive compounds but also by the structural development of different tissues [34]. In the present study, the radial proportion of RB initially increased with growth age and subsequently declined, reaching its maximum value (16.67%) in five-year-old plants. Although the concentrations of NC and CHE were highest in the RB of three-year-old plants, the radial proportion of RB at this stage remained relatively low (10.86%). By contrast, five-year-old plants exhibited the highest radial proportion of RB together with a second peak in MAG accumulation.

As secondary growth proceeds, continuous cambial activity promotes xylem development, resulting in an increasing radial proportion of RW. Because RW contains only low concentrations of the quantified bioactive compounds, expansion of this tissue may reduce the overall concentration of these constituents in whole-root medicinal materials, even when their concentrations in RB remain relatively stable [17,35,36]. Similar dilution effects associated with tissue development have also been reported in several perennial medicinal plants, including *Paeonia* spp. and *Astragalus* spp. [2,35,36].

Taken together, these findings indicate that both tissue proportion and metabolite concentration should be considered when evaluating the chemical quality of cultivated *Z. nitidum* [2]. Although alkaloid concentrations reached their maximum in three-year-old plants, the higher radial proportion of RB in five-year-old plants may contribute to a higher accumulation of bioactive constituents at the whole-root level. Therefore, further studies integrating tissue biomass and metabolite yield will be necessary to determine the optimal harvesting age under field cultivation conditions. Additionally, as the present study evaluated samples harvested within a single year, long-term tracking across consecutive seasons will be valuable to fully elucidate the influence of inter-annual environmental fluctuations, despite macro-environmental variables having been controlled via standardized concurrent sampling.

## 5. Conclusions

This study systematically characterized the spatial distribution and age-dependent accumulation patterns of four major bioactive compounds in the roots of cultivated *Z. nitidum*. The results demonstrated pronounced tissue specificity, with all four compounds being preferentially accumulated in RB, whereas RW, which accounted for more than 80% of the root radial proportion, contained only trace amounts of the quantified constituents. These findings indicate that RB is the principal tissue contributing to the chemical quality of *Z. nitidum* medicinal materials. Growth age significantly affected the accumulation of the investigated compounds. NC and CHE reached their highest concentrations in the RB of three-year-old plants, whereas the highest radial proportion of RB was observed in five-year-old plants. These tissue-level trends provide preliminary insights into potential harvesting strategies based on specific target compounds. For instance, three-year-old roots may be preferred when prioritizing maximum concentrations of benzophenanthridine alkaloids, whereas five-year-old plants exhibit a favorable tissue ratio. However, because overall plant biomass and total metabolite yield were not measured in this study, these age-related patterns should be interpreted cautiously and cannot serve as definitive determinations of the optimal commercial harvesting age. Overall, this study provides a scientific basis for optimizing tissue-specific processing, quality evaluation, and sustainable utilization of cultivated *Z. nitidum* resources.

## Data Availability

The original contributions presented in this study are included in the article/Supplementary Material. Further inquiries can be directed to the corresponding authors.

## Supplementary Materials

The following supporting information can be downloaded at: https://www.mdpi.com/article/10.3390/plants15203101/s1, Figure S1: Standard curves of four active compounds in *Zanthoxylum nitidum*. (a) Magnoflorine (MAG); (b) Hesperidin (HES); (c) Nitidine chloride (NC); (d) Chelerythrine (CHE).
