# Supplementary material for: Spatiotemporal Accumulation of Four Major Bioactive Compounds in Zanthoxylum nitidum Roots: Implications for Tissue-Specific Processing and Harvesting Strategies
Source: Plants (Basel). 2026 Oct 10;15(20):3101. doi: 10.3390/plants15203101 (PMC13709119; doi:10.3390/plants15203101)
Supplement: Supplementary file 1 [file plants-15-03101-s001.zip › plants-4606410-supplementary.pdf]

Spatiotemporal Accumulation of Four Major Bioactive Compounds in *Zanthoxylum nitidum* Roots: Implications for Tissue-Specific Processing and Harvesting Strategies

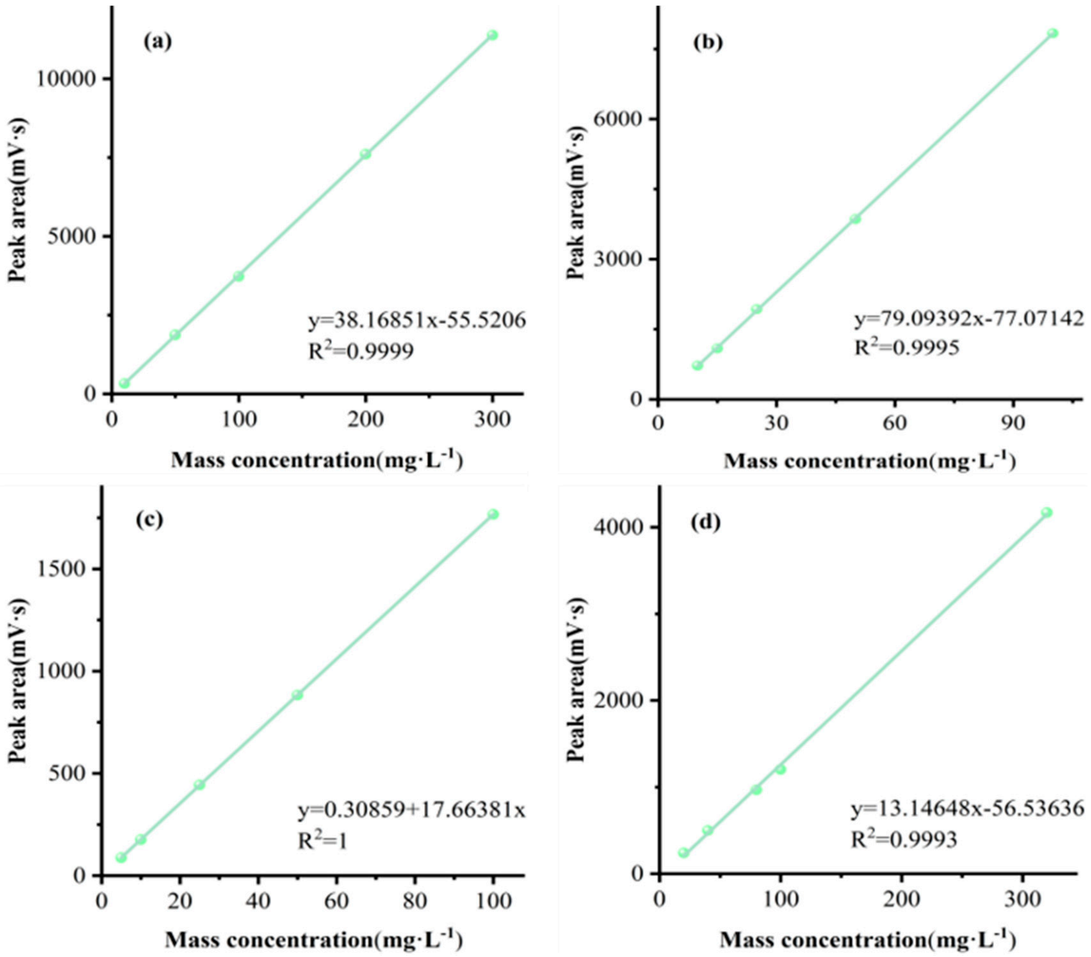

Figure S1. Standard curves of four active compounds in *Zanthoxylum nitidum*. (a) Magnoflorine (MAG); (b) Hesperidin (HES); (c) Nitidine chloride (NC); (d) Chelerythrine (CHE).
